# Dysprosium Doped Zinc Oxide for NO_2_ Gas Sensing

**DOI:** 10.3390/s22145173

**Published:** 2022-07-10

**Authors:** Ghada El Fidha, Nabila Bitri, Sarra Mahjoubi, Fatma Chaabouni, Eduard Llobet, Juan Casanova-Chafer

**Affiliations:** 1 École Nationale Supérieure d’Ingénieurs de Tunis, Université de Tunis, Avenue Taha Hussein Montfleury, Tunis 1008, Tunisia; elfidhaghada93@gmail.com; 2 Laboratoire de Photovoltaïque et Matériaux Semi-Conducteurs, École Nationale d’Ingénieurs de Tunis, Université de Tunis, Tunis 1002, Tunisia; nabila.b.hamdi@gmail.com (N.B.); mahjoubi.sa@gmail.com (S.M.); fatma.chaabouni@enit.utm.tn (F.C.); 3 Microsystems Nanotechnologies for Chemical Analysis (MINOS), Universitat Rovira i Virgili, Avda. Països Catalans, 26, 43007 Tarragona, Spain; eduard.llobet@urv.cat

**Keywords:** Dy-doped ZnO, gas sensor, NO_2_, humidity

## Abstract

Pure and dysprosium-loaded ZnO films were grown by radio-frequency magnetron sputtering. The films were characterized using a wide variety of morphological, compositional, optical, and electrical techniques. The crystalline structure, surface homogeneity, and bandgap energies were studied in detail for the developed nanocomposites. The properties of pure and dysprosium-doped ZnO thin films were investigated to detect nitrogen dioxide (NO_2_) at the ppb range. In particular, ZnO sensors doped with rare-earth materials have been demonstrated as a feasible strategy to improve the sensitivity in comparison to their pure ZnO counterparts. In addition, the sensing performance was studied and discussed under dry and humid environments, revealing noteworthy stability and reliability under different experimental conditions. In this perspective, additional gaseous compounds such as ammonia and ethanol were measured, resulting in extremely low sensing responses. Therefore, the gas-sensing mechanisms were discussed in detail to better understand the NO_2_ selectivity given by the Dy-doped ZnO layer.

## 1. Introduction

According to the World Health Organization, around 91% of the world’s population lives under low air-quality levels, which causes the premature death of about seven million people worldwide every year [1]. Therefore, this exposure to harmful gases is a major challenge that should be addressed. Within the different air pollutants, nitrogen dioxide (NO_2_) represents a potential threat to human health and the environment [2]. This gas is usually released during the combustion of fossil fuels, automobile exhaust, and factory processes. In addition, long-term exposure to moderate concentrations of NO_2_ can significantly increase the incidence of acute respiratory diseases such as asthma and bronchitis [3,4].

From this perspective, the development of gas sensors with high sensitivity and selectivity is considered an essential action for human health protection and industrial development. To date, pollutant levels can be detected through a wide variety of techniques such as gas chromatography or electrochemical cells [5,6]. However, some drawbacks such as the impossibility of monitoring the gases in real-time or their associated high costs are preventing their implementation in commercial devices. Conversely, metal oxide semiconductor gas sensors (MOXs) have attracted great attention owing to their outstanding sensitivity, easy fabrication, suitability for miniaturization, and inexpensiveness. In this regard, zinc oxide (ZnO) is one of the most popular n-type MOXs. This semiconductor exhibits outstanding properties such as high electron mobility [7], large chemical and thermal stability under different operating conditions [8], and low toxicity. ZnO is widely used for gas sensing applications, and the development of different nanostructures can modify the gas-sensing properties to some extent [9,10].

However, sensing devices composed of pure ZnO usually present limited specificity to gas compounds [11]. In this sense, the use of additional nanomaterials for decorating or doping ZnO is a widely adopted strategy to improve sensing properties such as selectivity and sensitivity or to reduce the optimum operating temperature [12,13,14]. Most of the research studies are centered on the use of alternative metal or metal oxide compounds for doping ZnO [15]. Nonetheless, the use of rare-earth materials for doping ZnO in gas sensing applications is still at a preliminary, exploratory phase. These elements present excellent physicochemical properties based on the electronic transitions occurring within the 4f energy shell [16]. As a result, their high conductivity, magnetic, electrochemical, and luminescent properties enabled their effective use as photocatalysts [17], photodetectors [18], Schottky diodes [19], and UV detectors [20], to cite only a few. Moreover, rare-earth compounds have been proved as promising candidates for improving gas sensing performance owing to their catalytic nature, fast oxygen ion mobility, and high surface basicity [21,22]. 

Among the various rare-earth elements, Dysprosium (Dy) has been used several times for improving sensing performance. For instance, Keerthana Bose et al. studied the effect of doping SnO_2_ with Dy, revealing higher sensing responses compared to those of pure SnO_2_ counterparts [23]. This superior sensitivity was related to the higher boundaries and catalytic sites of Dy, resulting in more efficient adsorption of oxygen. As a result, the adsorbed oxygen traps electrons from the conduction band of the semiconductor, inducing a larger resistance change and additional reactive sites [23]. In this context, G. Singh et al. also doped SnO_2_ nanostructures with Dy for demonstrating that the optimal working temperature could be lowered, reducing the power consumption of the sensing device [24]. In addition, G. Singh et al. reported selective detection of ethanol when employing Dy-doped SnO_2_, which was attributed to the increased number of oxygen vacancies and higher surface area of the doped nanoparticles [24]. Not limited to this, K. Anand et al. studied the detection of several gases such as methanol, ethanol, acetone, and ammonia using In_2_O_3_ doped with Dy^3+^ nanoparticles [25]. The sensing performance was improved when the MOX was doped with a 10% Dy, probably because of the higher number of oxygen vacancies/defects, high surface basicity, and large lattice distortion induced by the dopant element [25]. 

Herein, we report for the first time the sensing properties under dry and humid environments of Dy-doped ZnO thin films deposited using the RF magnetron reactive sputtering technique. The use of this rare-earth nanomaterial as a dopant element enables the distortion of the ZnO lattice, resulting in the creation of structural defects related to oxygen vacancies that enhance sensing performance. Besides, the structural, compositional, morphological, optical, and electrical properties of the prepared thin films were investigated. Finally, the sensing properties of the pure and Dy-doped ZnO were evaluated towards different gases such as nitrogen dioxide (NO_2_), ammonia (NH_3_), and ethanol (C_2_H_6_O).

## 2. Materials and Methods

### 2.1. Material Synthesis

The radiofrequency (RF) magnetron sputtering technique was used to fabricate pure and Dy^3+^ doped (6 wt.%) ZnO films on glass and alumina substrates at room temperature. ZnO and Dy_2_O_3_ powders were mixed for 10 min to produce a uniform mix. The Dy-doping content was adjusted at 0 and 6 wt.%. The mix of the powders was lightly tamped on the backing plate to produce a uniform thickness. After that, the vacuum chamber was evacuated before the deposition to 10^−4^ Pa to eliminate the contaminations using a diffusion pump. The target-to-substrate distance was 65 mm and the substrates were rotated at 15 rad/min. The magnetron sputtering power was established to 200 W, and 12.5 sccm of argon flow rate was applied. The deposition time was fixed to 3 h. It is worth highlighting the outstanding reproducibility between batches of samples through magnetron sputtering synthesis protocols. 

### 2.2. Material Characterization Techniques

The structural characterization of the thin films was evaluated using XRD (X-ray diffraction) with a Siemens D5000 diffractometer. The angular 2θ diffraction range was between 20 and 70°. Their optical properties were analyzed using a Shimadzu-UV1800 spectrophotometer. Field emission scanning electron microscopy (FESEM) was used to study the surface morphology. The roughness of samples, given by the surface root mean square (RMS) was analyzed using atomic force microscopy (AFM) (Dimension Icon, Bruker, Billerica, MA, USA) in intermittent contact mode and the data analysis was performed using the WSxM (version 5.0 Develop 6.4 software), which is a freeware (Madrid, Spain). The compositional investigation was conducted using energy-dispersive X-ray spectroscopy (EDS). Finally, the electrical properties of the prepared samples were determined by impedance spectroscopy using a Hewlett-Packard 4192 analyzer within the (1–13,000 kHz) frequency range. The configuration for electrical measurements was made using two electrodes, which were painted on the two extremities of the thin film using a conductive silver paste. The wired samples were placed inside a furnace for performing impedance spectroscopy studies at different temperatures.

### 2.3. Gas Sensing Tests

**Figure 1:**
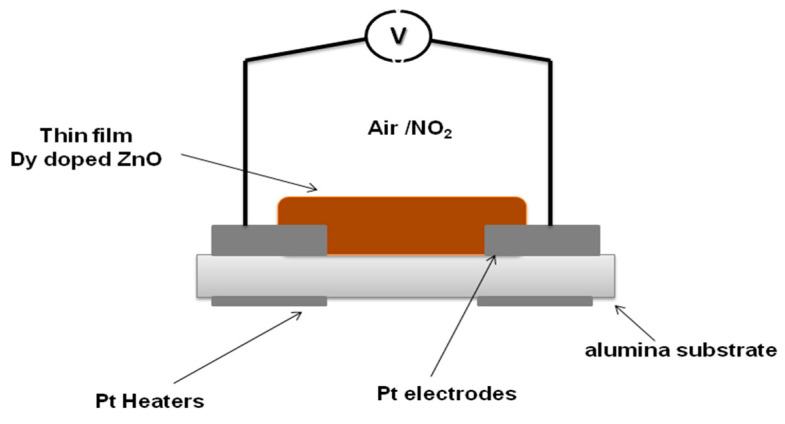
Structure of the gas sensing device.

With the aim of studying and comparing the gas sensing properties of pure and Dysprosium doped ZnO thin films, the sensitive films were deposited onto commercial alumina substrates (0.4 × 2.25 cm in size, see Figure 1). The sensing devices comprise 0.25 × 0.73 cm screen-printed interdigitated platinum electrodes and a heating resistor (6 Ω) on the backside (Figure S1). The resulting gas sensors were placed in an airtight Teflon chamber and exposed to different concentrations of the target gases. The electrical resistance was measured using an Agilent-34972A multimeter and the operating temperature was modulated by an external power supply. Several NO_2_ gas concentrations at the ppb range were measured in dry and humid (50% of relative humidity) environments. 

The gas sensing performance of these sensors was tested at various operating temperatures (25 °C, 100 °C, 150 °C and 200 °C) when exposed to different concentrations of NO_2_ (250, 500, 750 and 1000 ppb). The gas exposure and recovery times were fixed at 15 and 30 min, respectively. The sensor responses were estimated by the following equation: response = [(R_g_ − R_a_)/R_a_] × 100 expressed in percentage. Where R_a_ corresponds to the resistance value in air, while R_g_ represents the resistance value obtained when the sensor was exposed to the target gas. Finally, the sensor selectivity was evaluated measuring NH_3_ and ethanol vapors. The total flow rate was kept constant at 100 mL/min throughout the measurements.

## 3. Results

### 3.1. Morphological and Compositional Characterization

**Figure 2:**
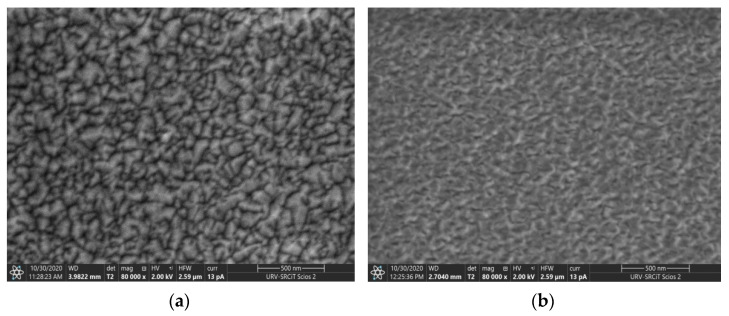
FESEM images of pure ZnO (**a**), and Dy@ZnO sample (**b**).

The FESEM analysis of pure and Dy-doped ZnO samples is shown in Figure 2. The surfaces are uniform, and dense distribution of grains can be observed, revealing highly homogeneous layers. The film thicknesses were estimated using the cross-section of SEM images (Figure S2), obtaining 490 nm and 230 nm for the undoped and doped ZnO, respectively. This divergence in the film thicknesses will be translated in different resistivity levels. However, considering that gas compounds would interact with the sensor surface, the film thickness in not directly affecting to the sensing performance.

AFM was employed to study the surface roughness (Figure S3). The analysis confirms the high density and homogenous characteristics of the films. Root mean square (RMS) roughness values of a 7.1 and 5.4 were obtained for Dy-doped and pure ZnO, respectively, revealing a smooth surface morphology. This slight decrease in surface roughness for the doped ZnO is in accordance with the FESEM images. As a result, the Dy@ZnO surface tends to show lower particle size and fewer agglomerations.

The compositional analysis was carried out using an Energy Dispersive X-Ray Spectroscopy (EDX) coupled to the FESEM equipment. Table 1 summarizes the weight percentage of the different elements, confirming the presence of Zn and O in the pure sample without impurities. In addition, the doped sample shows an experimental Dy content (5.6 wt.%) similar to the theoretical content (6 wt.%)) according to the synthesis protocol. Thereby, this analysis confirms the presence of Dy, resulting in suitable doping of ZnO thin films.

### 3.2. Structural Characterization

**Figure 3:**
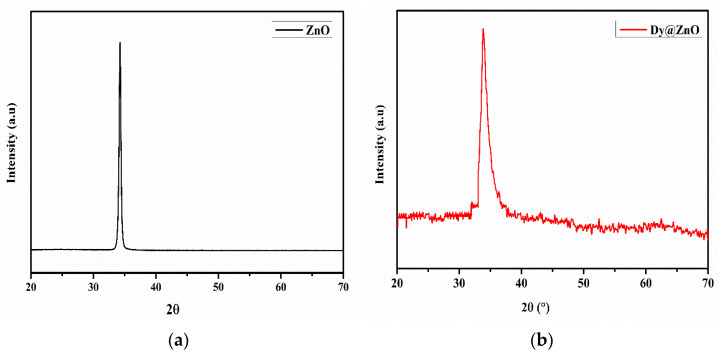
X-ray diffraction pattern of undoped ZnO thin film (**a**) and Dy-doped ZnO (**b**).

The synthesized thin films were also studied using X-ray diffraction (XRD). Figure 3 shows the XRD patterns for both samples, revealing a preferential peak (002) at 2θ = 34.3°, corresponding to the hexagonal wurtzite structure (JCPDS card no. 36-1451). No additional diffraction peaks corresponding to Dy or Dy_2_O_3_ can be observed, which indicates the suitable incorporation of Dy atoms into the ZnO lattice. In addition, a small peak shift is observed towards the lower angles from 34.26° to 34.08°. This is due to the substitution of the doped Dy^3+^ ions in Zn^2+^ sites. Furthermore, a peak shift occurs when a dopant of a larger ionic radius (0.91 Å) is substituted in the place of a host with a smaller ionic radius (0.74 Å) [26].

In fact, according to Vegard’s rule [27], the substitutional incorporation of a cation having a larger ionic radius than the host cation will lead to the expansion of the lattice. As a result, the incorporation of Dy^3+^ ions as substituents at the Zn^2+^ sites is more likely than at interstitial positions [28].

The crystallite size D was calculated using the Debye–Scherrer formula [29]:

(1) $$D=\frac{k\lambda }{\beta \cos\theta }$$

where k = 0.9, λ is a wavelength of 0.154 nm, β is full-width half maxima, and θ is the diffraction angle in radians. Table 2 summarizes the results obtained, in which a decrease in crystallite size and increase in FWHM is observed when the ZnO is doped with Dy. This is probably due to the exchange of Zn^2+^ by Dy^3+^ ion in the films [30]. Indeed, Dysprosium atoms can affect the coalescence process and prevent the formation of larger grains, lowering the surface roughness as observed in the AFM analysis.

### 3.3. Optical Characterization

**Figure 4:**
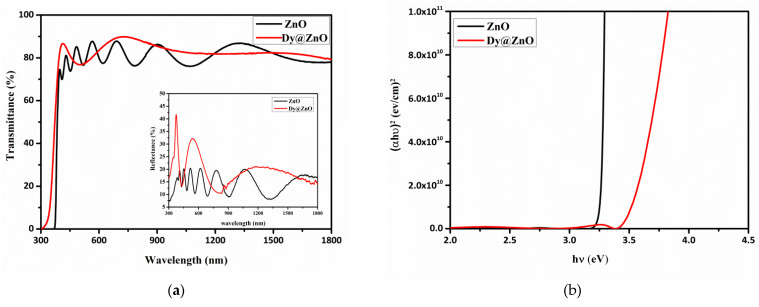
Optical transmittance and reflectance spectra for the Dy-doped ZnO thin films (**a**). The plot of (αhν)^2^ versus (hν) of undoped and Dy-doped ZnO thin films (**b**).

Figure 4a shows the transmittance and reflectance curves for the pure and Dy-doped ZnO thin films. The interference fringes confirm the homogeneity and the excellent surface quality of the deposited films. The interference fringes reveal the aspect of the surface, which is a smooth reflection that enables the achievement of no scattering losses. It is worth mentioning that the samples have a high optical transmittance, achieving 80%, and a fundamental absorption edge at 380 nm. The reflectance spectra show low values of reflectance for both films.

The optical band gaps were estimated by Tauc’s equation [31]:

(2) $${\left(\alpha h\nu \right)}^{2}=A\left(h\nu -E_{g}\right)$$

(3) $$\alpha =\frac{1}{d}\left(\frac{\left(1-R^{2}\right)}{T}\right)$$

where ν is the frequency of the incident photons, hν is the photon energy, A is a constant (0.9) that corresponds to the probability parameter for the transitions, which measures the disorder of the material, and α is the absorption coefficient. Then, the bandgap energy was estimated by extrapolation of the linear portion of (ahν)^2^ versus photon energy (hν). Figure 4b shows (αhν)^2^ as a function of photon energy for both samples. As a result, a bandgap of 3.54 eV and 3.26 eV was obtained for doped and undoped ZnO, respectively. This slight band gap broadening can be related to the Burstein-Moss effect [32]. This phenomenon is based on the increase of the carrier concentration and the filling of the lower states in the conduction band for n-type materials, enabling the shift of the Fermi level towards the conduction band [33].

In addition, the photoluminescence is an effective way to obtain information about the intrinsic defects of ZnO. Pure ZnO and Dy-doped thin films were analyzed using PL in our previous work [17]. We observed two main peaks, one in the UV at around 380 nm and a broader emission band in the visible range from 450 to 700 nm. The presence of this second peak in the visible range is attributed to defects which could be related to oxygen vacancies, which are strongly related with outstanding interactions with gas compounds [34]. Thereby, the incorporation of Dy atoms into the ZnO lattice tends to enhance the sensing performance owing to the creation of defects that will act as reactive sites. M. Salah and collaborators developed a ZnO-doped rare-earth lithium sensor, demonstrating an improvement in the performance for detecting ethanol when ZnO was doped. According to the authors, the creation of defects by the incorporation of Li+ ions into the ZnO network and the increase in oxygen vacancies result in a higher reactivity with gases due to the higher density of active sites [35]. P. Bharathi et al. doped ZnO with gadolinium (Gd) to detect xylene. As a result, the Gd-doped ZnO showed higher responses (4-fold) than pure ZnO [36].

In the same context, Kumar et al. developed ZnO-based gas sensors doped with Erbium (Er). The rare-earth-doped films exhibited better stability, reproducibility, and sensing responses (up to 3-fold) than the undoped pure ZnO films. This better performance was also attributed to the presence of Er ions since their incorporation into the ZnO lattice creates defects in the host structure. Thereby, the rare-earth ions and the defects created by their incorporation (i.e., oxygen vacancies) act as gas adsorption sites, enhancing the sensing performance [34].

### 3.4. Electrical Characterization 

**Figure 5:**
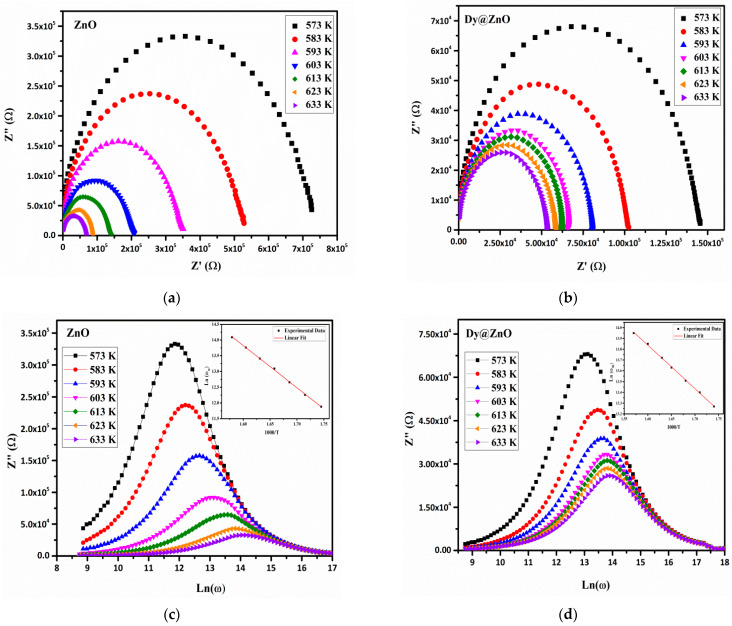
Complex impedance spectra at different temperatures for the pure (**a**) and Dy-doped ZnO (**b**) thin films. Angular frequency dependence of Z” at different temperatures for the pure (**c**) and Dy-doped ZnO (**d**) thin films.

The complex impedance curves of Z” as a function of Z’ regarding pure and doped ZnO thin films in the temperature range (573–633 K) are displayed in Figure 5a,b. The data analysis presents only one semi-circle, which indicates that the electrical process in the samples is mainly due to the grain contribution. Each semi-circle can be modeled by an electrical equivalent circuit, which consists of a resistance (R) and a capacitance (C) connected in parallel. It is worth noting that the radius of the semicircular arcs decreases as the temperature increases for the two samples, revealing that the electrical conductivity is thermally activated, as well as the relaxation time distribution [37]. 

Moreover, the resistivity levels (i.e., baseline resistance) of the Dy-doped ZnO are lower than those obtained for the pure ZnO. This is probably because of the partial substitution of Zn^2+^ in the pure sample by Dy^3+^ in the doped thin film and the thinner film. As a result, the additional free electrons are released into the conduction band, increasing the electrical conductivity [13]. In order to study the relaxation time as a function of temperature, the variation in the imaginary part of impedance Z” versus angular frequency at different temperatures of ZnO undoped and doped thin films are presented in Figure 5c,d. The analysis shows that all the spectra have a single peak of relaxation for each temperature whose maximum (Z” max) shifts to higher frequencies with increasing temperatures [38].

**Figure 6:**
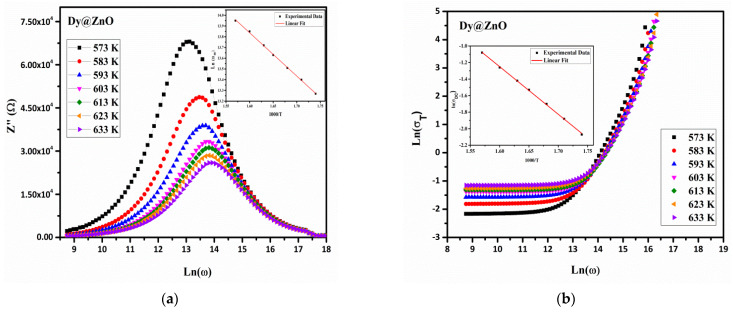
Angular frequency dependence of $\sigma_{T}$ conductivity at different temperatures for the pure (**a**) and Dy-doped (**b**) ZnO thin films.

The variation in Ln ($\sigma_{T}$) as a function of Ln (ω) at different temperatures is shown in Figure 6. At low frequencies, the conductivity is almost independent of the frequency studied, which is attributed to the contribution of the DC current. Conversely, at higher frequencies, the conductivity increases with the frequency applied, which is in agreement with Jonscher’s Law [39]:

(4) $$\sigma_{AC}\left(\omega \right)=A\omega^{s}$$

where *A* is a complex proportionality constant, ω is the angular frequency, and *S* is the exponent in the range of 0 < *S* < 1. The variation in Ln ($\sigma_{DC}$) as a function of the inverse of the temperature 1000/T is shown in the inset of Figure 6, and the obtained activation energy values are summarized in Table 3. The values of activation energies deduced from both DC conductivity and the frequency are in good agreement, demonstrating that the conduction process is related to the same mechanism. 

The relaxation frequency obeys the Arrhenius law [40]:

(5) $$\omega_{m}=\omega_{0}e^{\frac{-E_{a}}{K_{B}T}}$$

where $\omega_{0}$ is a constant, *k_B_* is the Boltzmann constant and *E_a_* is the thermal activation energy of the carriers’ charge. The activation energy is considered an essential parameter to evaluate sensing performance. In other words, the activation energy can be explained as the thermal energy required to excite electrons and their movement from the valence to the conduction band. Therefore, a lower value of activation energy enables an easier overtake of the energy barrier when interacting with gas compounds, and consequently, higher sensitivity can be expected [41]. 

### 3.5. Gas Sensing Measurements 

The gas sensing properties of the developed sensitive thin films were evaluated through the changes in their resistance when exposed to the target gases. The sensing performance of both layers was first assessed towards different operating temperatures. Specifically, successive pulses of 1 ppm of NO_2_ were applied at 25, 100, 150, and 200 °C for establishing the optimal working conditions (Figure S4). As a result, 150 °C has been demonstrated as the best operating temperature to detect NO_2_ for both gas sensors. This optimum operating temperature agrees with previously reported ZnO-based sensors [42,43,44]. At low temperatures, the sensing responses are usually limited because the thermal energy is not enough for activating the metal oxide properties. However, the sensor response will be improved when increasing the temperature, owing to the higher activity of oxygen ions at the ZnO surface and favoring the interaction with gas compounds. From this perspective, when detecting NO_2_ at 150 °C, the activation barrier is probably lowered, enabling an enhancement in the rate of gas adsorption on the surface of the semiconductor. As a result, the gas sensor operated at 150 °C showed the highest sensor response experimentally. Nevertheless, when the operating temperature rises above this optimum value, the electrical responses will begin to decrease [45]. This is due to the probably lower adsorption rate of gas compounds in comparison to the promoted desorption rate at 200 °C [46].

**Figure 7:**
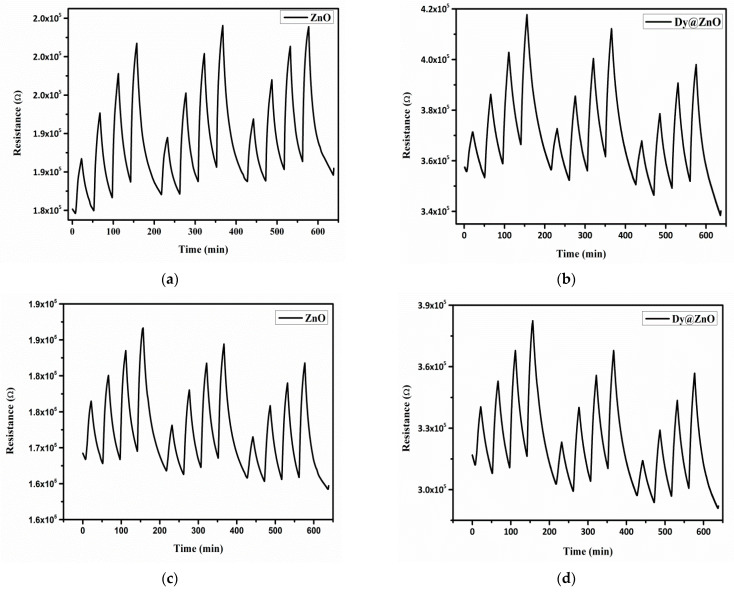
Sensor resistance when detecting NO_2_ at 150 °C with pure (**a**) and Dy-doped ZnO (**b**) in dry conditions. The experiments were repeated under a humid atmosphere for pure (**c**) and Dy-doped ZnO (**d**) thin films.

**Figure 8:**
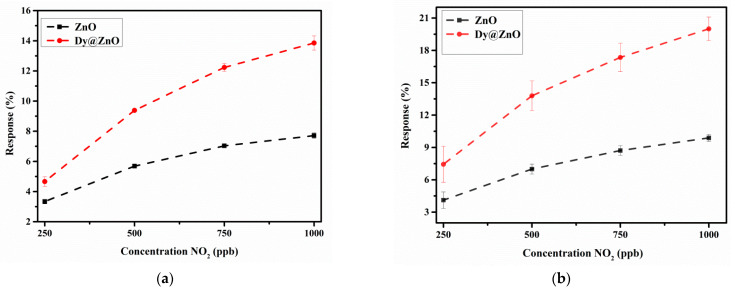
The response of the sensors as a function of concentration NO_2_ in dry conditions (**a**) and humid conditions (**b**). The sensor responses were defined as ∆R/R_a_ expressed in percentage.

Thereby, once the optimum operating temperature was established, repeated exposure and recovery cycles were applied, namely 250, 500, 750, and 1000 ppb of NO_2_. Figure 7a,b show the sensor resistance for the bare ZnO and the Dy@ZnO samples, respectively. Both sensors showed significant response, stability, and repeatability. However, Figure 8a depicts the resulting calibration curves, revealing that Dy-doped ZnO shows higher responses (2-fold) than the pure ZnO. The calibration curves were fitted using a linear regression model, and their respective slopes were used for estimating the sensitivities. As a result, since the sensor sensitivity is given by the slope of the calibration curves, it was observed an increase from 5.71 × 10^−3^ for the pure ZnO to 1.21 × 10^−2^ for the doped ZnO This better sensing performance when ZnO was doped with Dy^3+^ is probably related to an excess of oxygen species adsorbed on the surface. The reason is the higher lattice distortion and a larger number of carriers created by the dopant, resulting in more oxygen ions adsorbed on the surface [47]. In addition, the moderate operating temperature applied helps achieving an improved long-term stability of the ZnO-based sensors [48].

Despite the noteworthy detection of NO_2_ using the Dy@ZnO nanohybrid under dry conditions, the assessment of the humidity effect is essential for practical applications in ambient monitoring [49]. Thereby, since the presence of water molecules is a well-known interferent, the previous experiments were reproduced under humid conditions (relative humidity was set to 50% @23 °C). Figure 7c,d show sensor resistance under humid conditions of pure and Dy-doped ZnO, respectively. Stable and repeatable responses were obtained in the presence of ambient moisture, demonstrating the robustness of the sensitive films for detecting NO_2_ in humid conditions. Figure 8b depicts the calibration curves obtained for both sensitive films. It is worth mentioning that higher resistance changes for pure and Dy-doped ZnO were obtained when detecting NO_2_ in a humid environment in comparison to a dry atmosphere. This is probably because water molecules tend to act as an electron-withdrawing specie as NO_2_. In that way, the simultaneous presence of both compounds probably enhances the overall resistance changes. However, considering the sensitivity is given by the slope of the calibration curves, both thin films showed slightly higher sensitivity in a humid environment in comparison to dry conditions (Table S1). Interestingly, in the presence of ambient moisture, the pure and Dy-doped ZnO showed a 17% and 28% increase in response when detecting NO_2_ compared to a dry environment, respectively. The presence of Dy increases the reactivity of the thin film, but without compromising the stability and repeatability of the gas detection.

**Figure 9:**
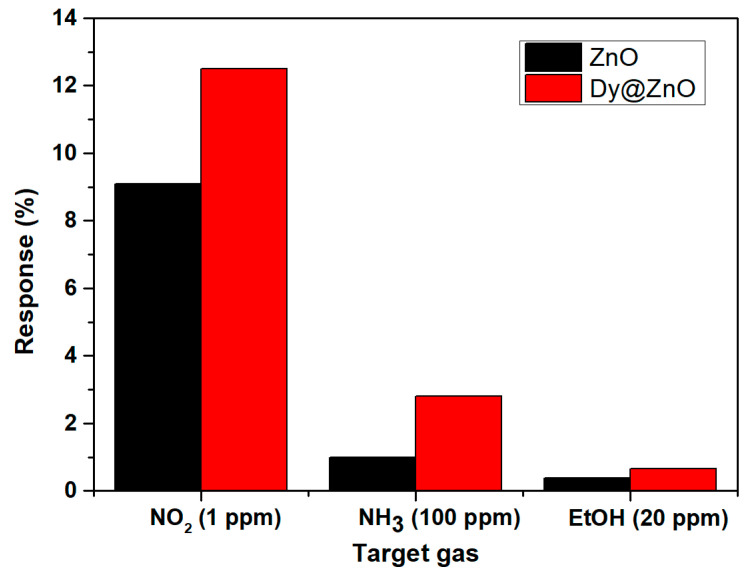
Comparison between pure and Dy-doped ZnO for different target gases in dry conditions.

Cross-sensitivity is a common problem in chemoresistors that frequently prevents their implementation in commercial devices. From this perspective, Figure 9 depicts an additional test summarizing a response comparison when detecting 1 ppm NO_2_, 100 ppm of NH_3,_ and 20 ppm of ethanol at 150 °C. An example of the sensing responses obtained for an electron-donor gas compound as NH_3_ is represented in Figure S5. It is worth noting that significantly higher responses for NO_2_ were obtained in comparison to other gases such as NH_3_ and ethanol, even though the lower concentration range tested for this gas. The reason is probably explained by previous experimental findings through density functional theory (DFT) calculations on ZnO nanostructures. The adsorption energy for NO_2_ is significantly lower than that of other gases tested such as NH_3_. In contrast, the charge transfer derived from this interaction is higher in the case of NO_2_ [50,51]. In other words, when Dy-doped ZnO is detecting NO_2_ levels, more dynamic adsorption–desorption processes and higher resistance changes may occur owing to the small binding energy and the large charge transfers associated to this interaction. 

Nevertheless, it is interesting to mention that even for gases with low responses such as NH_3_ and ethanol, the Dy-doped sample shows higher sensing responses than the pure ZnO. Overall, the substitution of Zn^2+^ ions by Dy^3+^ atoms creates more active sites for interacting with gases, which probably enhance the response towards a wide variety of gases in comparison to their undoped ZnO. Moreover, since this rare-earth dopant usually presents a 3+ oxidation state, its incorporation into the ZnO lattice induces an excess of free electrons [52]. This higher density of free electrons is probably another reason for explaining the effective interaction with an electron-withdrawing gas such as NO_2_. The cross-sensitivity test depicted in Figure 9 was conducted under dry conditions. However, considering the significant difference in the sensing responses obtained when detecting NO_2_ in comparison to the other gases, higher NO_2_ sensing performance towards NH_3_ or ethanol can be also expected under variable humidity levels.

## 4. Conclusions

Pure and Dy-doped ZnO thin films were synthesized by RF magnetron sputtering technique. XRD patterns of the elaborated films present the hexagonal wurtzite structure with a preferential orientation along [2] direction. According to the SEM micrographs, all structures have a uniform, homogeneous, and densely packed microstructures. Doping ZnO with dysprosium leads to higher sensing responses and the effect of the relative humidity was also studied. Accordingly, a stable, sensitive, and repeatable NO_2_ detection was achieved employing Dy@ZnO as the active layer. In addition, sensitivity remains almost invariable under dry and humid atmospheres, paving the way towards the use of doped MOX chemoresistors for ambient monitoring purposes.

## Figures and Tables

**Table 1:** The weight percentage of elements in pure and Dy-doped ZnO thin films.

	Weight Percentage (%)		
Sample	Zn	O	Dy
ZnO	61.35	38.65	0
Dy@ZnO	59.11	35.28	5.61

**Table 2:** Structural parameters of pure and Dy-doped ZnO thin films.

Sample	Pure ZnO	Dy@ZnO
2θ (degree)	34.5	33.8
β	0.27	0.34
D (nm)	30.8	24.1

**Table 3:** The activation energy of the pure and Dy-doped ZnO layers.

**Sample**	**Ea (ω_m_) (eV)**	**Ea (DC) (eV)**
ZnO	1.15	1.25
Dy@ZnO	0.34	0.45

## Data Availability

Data can be obtained from the authors upon request.

## Supplementary Materials

The following supporting information can be downloaded at: https://www.mdpi.com/article/10.3390/s22145173/s1, Figure S1: top and bottom view of the alumina substrate employed. Figure S2: Cross-section of the pure and Dy-doped ZnO thin films; Figure S3: AFM topography for pure (a) and Dy-doped (b) ZnO thin films; Figure S4: Response of pure and Dy-doped ZnO thin films towards 1 ppm of NO_2_ at different operating temperatures, ranging from 25 °C to 200 °C. Table S1: Sensitivity values obtained for the pure and Dy-doped ZnO under a dry and a humid atmosphere; Figure S5: Electrical responses when detecting NH3 at 150 °C.
