# Supplementary material for: Dysprosium Doped Zinc Oxide for NO2 Gas Sensing
Source: Sensors (Basel). 2022 Jul 10;22(14):5173. doi: 10.3390/s22145173 (PMC9317177; doi:10.3390/s22145173)
Supplement: Supplementary file 1 [file sensors-22-05173-s001.zip › sensors-1787404-supplementary.pdf]

Dysprosium Doped Zinc Oxide for NO_2 Gas Sensing

G. El Fidha[1,2], N. Bitri[2], S. Mahjoubi[2], F. Chaabouni[2], E. Llobet[3] and J. Casanova-Chafer*[3]

Address: [1]Université de Tunis, École nationale supérieure d'ingénieurs de Tunis, Avenue Taha Hussein Montfleury, 1008 Tunis, Tunisie, [2]Ecole Nationale d'Ingénieurs de Tunis, Laboratoire de Photovoltaïque et matériaux semi-conducteurs, 1002 Tunis, Tunisie and [3]MINOS, Universitat Rovira i Virgili, Avda. Països Catalans, 26, 43007, Tarragona, Spain

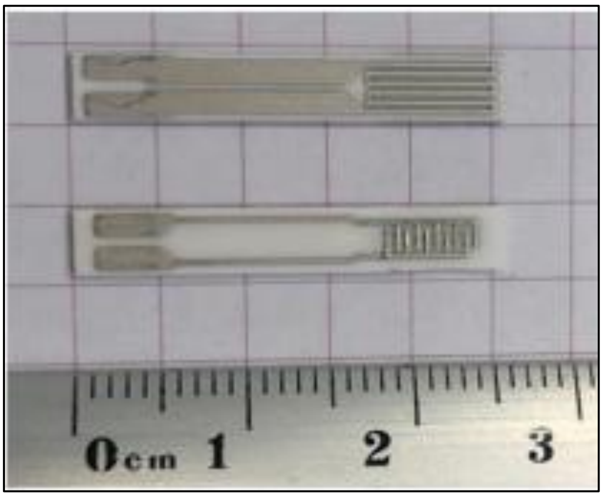

Figure S1. Top and bottom view of the alumina substrate employed.

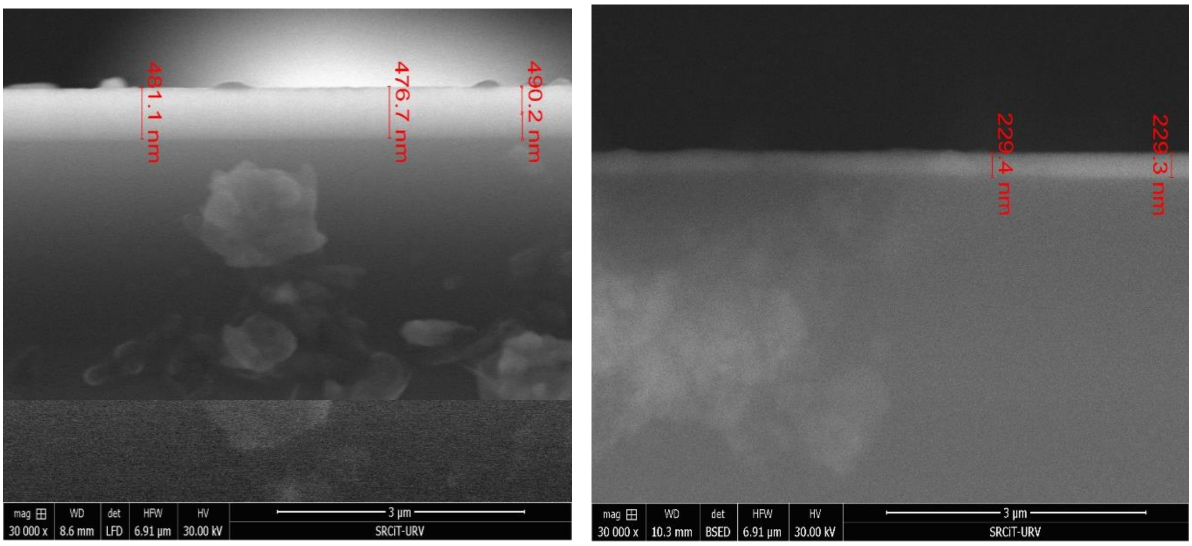

Figure S2. Cross-section of the pure ZnO thin film (left side) and Dy doped ZnO doped (right side).

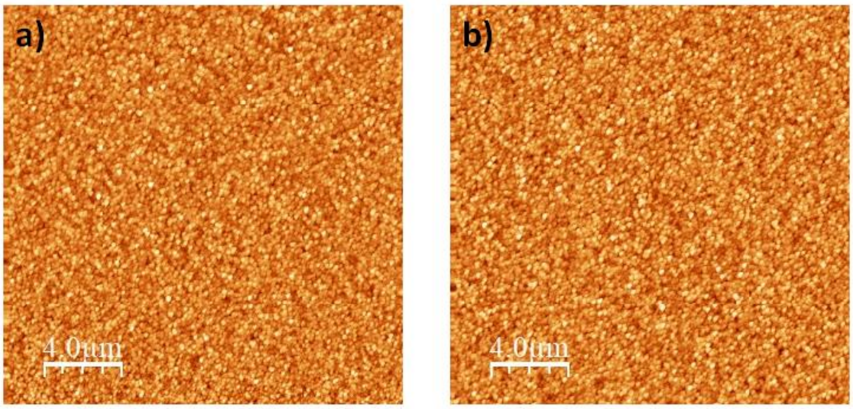

Figure S3. AFM topography for a) pure and b) Dy doped ZnO thin films.

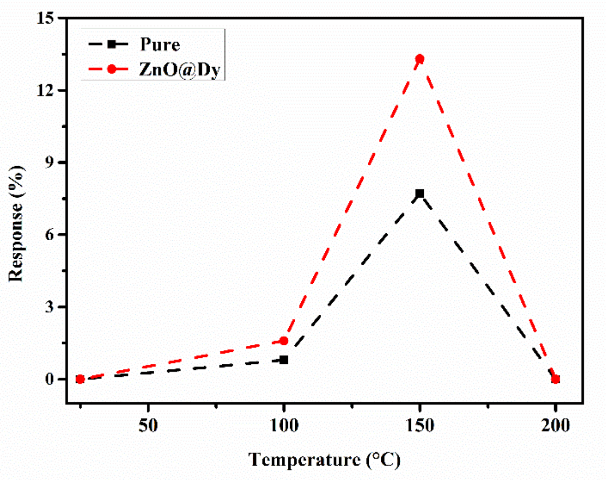

Figure S4. Response of pure and Dy-doped ZnO at 6% films towards 1 ppm NO_2 at different operating temperatures ranging from 25°C to 200°C in dry conditions.

Table S1. Sensitivity values of the pure and Dy doped ZnO under humidity and dry air.

Sensors	ZnO	ZnO@Dy
Dry air	$5.73\ 10^{-3}$	$1.21\ 10^{-2}$
humidity	$6.73\ 10^{-3}$	$1.56\ 10^{-2}$

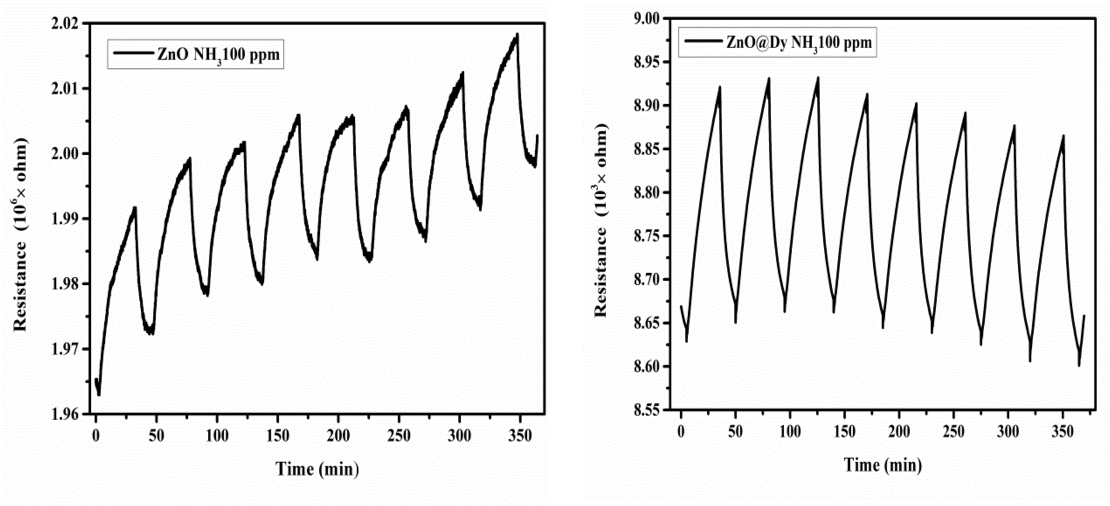

(a) (b)

Figure S5. Resistance changes of pure and Dy doped ZnO at 6 wt.% gas sensors upon exposure to 100 ppm of NH_3 at 150 ºC in dry conditions.
